# The Relevance of Circadian Clocks to Stem Cell Differentiation and Cancer Progression

**DOI:** 10.3390/neurosci3020012

**Published:** 2022-03-29

**Authors:** Astha Malik, Shreya Nalluri, Arpan De, Dilshan Beligala, Michael E. Geusz

**Affiliations:** 1 Division of Gastroenterology, Hepatology and Nutrition, Cincinnati Children’s Hospital Medical Center, Cincinnati, OH 45229, USA; shreya.nalluri@cchmc.org; 2 Department of Neurosurgery and Brain Tumor Center, Unit 1004, University of Texas MD Anderson Cancer Center, 1515 Holcombe Boulevard, Houston, TX 77030, USA; ade8@mdanderson.org; 3 Department of Molecular Biology and Biotechnology, University of Peradeniya, Peradeniya 20400, Sri Lanka; dbeligala@gmail.com; 4 Department of Biological Sciences, Bowling Green State University, Bowling Green, OH 43403, USA; mgeusz@bgsu.edu

**Keywords:** circadian rhythm, embryonic stem cell, neural stem cell, cancer stem cell, oligodendrocyte progenitor cell, differentiation, epithelial–mesenchymal transition, circulating tumor cell, metastasis, tumorsphere, glioma

## Abstract

The molecular mechanism of circadian clocks depends on transcription-translation feedback loops (TTFLs) that have known effects on key cellular processes. However, the distinct role of circadian TTFLs in mammalian stem cells and other less differentiated cells remains poorly understood. Neural stem cells (NSCs) of the brain generate neurons and glia postnatally but also may become cancer stem cells (CSCs), particularly in astrocytomas. Evidence indicates clock TTFL impairment is needed for tumor growth and progression; although, this issue has been examined primarily in more differentiated cancer cells rather than CSCs. Similarly, few studies have examined circadian rhythms in NSCs. After decades of research, it is now well recognized that tumors consist of CSCs and a range of other cancer cells along with noncancerous stromal cells. The circadian properties of these many contributors to tumor properties and treatment outcome are being widely explored. New molecular tools and ones in development will likely enable greater discrimination of important circadian and non-circadian cells within malignancies at multiple stages of cancer progression and following therapy. Here, we focus on adult NSCs and glioma CSCs to address how cells at different stages of differentiation may harbor unique states of the molecular circadian clock influencing differentiation and cell fate.

## 1. Introduction

### 1.1. The Master Circadian Pacemaker in the SCN

The suprachiasmatic nucleus (SCN) is a bilateral structure located in the anterior hypothalamus that receives photic signals through the optic nerve [1]. It is considered the central circadian clock independently regulating rhythms while also controlling the timing of subordinate circadian clocks inside and outside the nervous system [2]. Circadian clocks located outside the SCN are considered peripheral clocks, and these include clocks in fully differentiated cells and any functioning in the stem cells. Ablation of the SCN results in loss of circadian locomotor, endocrine, feeding, and drinking rhythms in rodents [3]. Transplantation of SCN tissue restores these rhythms, and the period length of the donor tissue determines the period of restored rhythms in the recipient animal [4]. This study and others showed that circadian clock properties are intrinsic to brain regions [5]. The SCN acts hierarchically and passes to the peripheral clocks its own timing signals along with external information from Zeitgebers, the daily cycles in the environment to which the circadian clocks entrain, thereby synchronizing the rhythms to the 24-h day.

Some peripheral clocks can function independently of the SCN such as in the liver, olfactory bulb, cornea, salivary gland, and various skin cell types including fibroblasts [6,7,8,9]. There are instances in which clocks of peripheral organs are not well synchronized with the oscillatory gene regulation of the SCN. Conflicting systemic signals, such as through feeding, can lead to a decoupling of peripheral clocks from synchronization with the central pacemaker. For example, when mice were fed during their rest phase, the liver clock shifted forward 12 h, while the SCN’s phase did not [10]. The strong relationships between nutrition, cell redox states, cancer, and the molecular circadian timing mechanisms have been described elsewhere [11].

Rhythms in particular peripheral circadian oscillators, including some in the brain, tend to dampen without frequent timing information from the circadian timing system [12], indicating that the peripheral clocks are able to integrate various physiological signals to mount appropriate rhythms in their tissues. Therefore, it has been proposed that mammals have a more centralized organization of circadian clocks when compared to nonmammalian vertebrates. In zebrafish, the peripheral clocks are directly light sensitive, whereas in mammals, the SCN is the master regulator of clock activity and entrainment to external light cycles [13,14].

The SCN regulates the phase of peripheral circadian clocks through endocrine and neural signals [15]. Serum levels of glucocorticoids, thyroid hormones, and several other ligands of nuclear receptors follow distinct circadian rhythms and are potential synchronizers of peripheral clocks to the SCN and to each other [16]. More recently, transforming growth factor-β (TGF-β) signaling has also been implicated in paracrine coupling of peripheral oscillators that maintains proper phase relationships [17]. Within neural tissue coupling through chemical and electrical synapses, cell adhesion proteins, and probably many other factors enable robust circadian rhythms [18]. Therefore, numerous signaling molecules may serve in complex timing interactions between single-cell circadian clocks, altering the period, phase, and amplitude of their rhythms.

The subdivision of the circadian timing system into subordinate interacting clocks is most justified where specific tissue samples have been harvested from organs and tested in vitro for evidence of intrinsic rhythmicity. This approach has been applied to nearly all major organs of rodents. Many individual cell types are also considered to be circadian clocks or at least oscillators, which is a more appropriate description when the output or input of timing information has not yet been identified. Essentially, a clock also contains “hands” expressing its phase along with a mechanism for readjusting its phase. Here, we will use “clock” to indicate a circadian oscillator, which can also be a collection of clocks, and has identified or suspected inputs and outputs. A “molecular clock” will refer to the mechanism of cellular clocks, which contains the clock “gears”.

### 1.2. The Role of TTFLs in the Molecular Clock Mechanism

**Figure 1:**
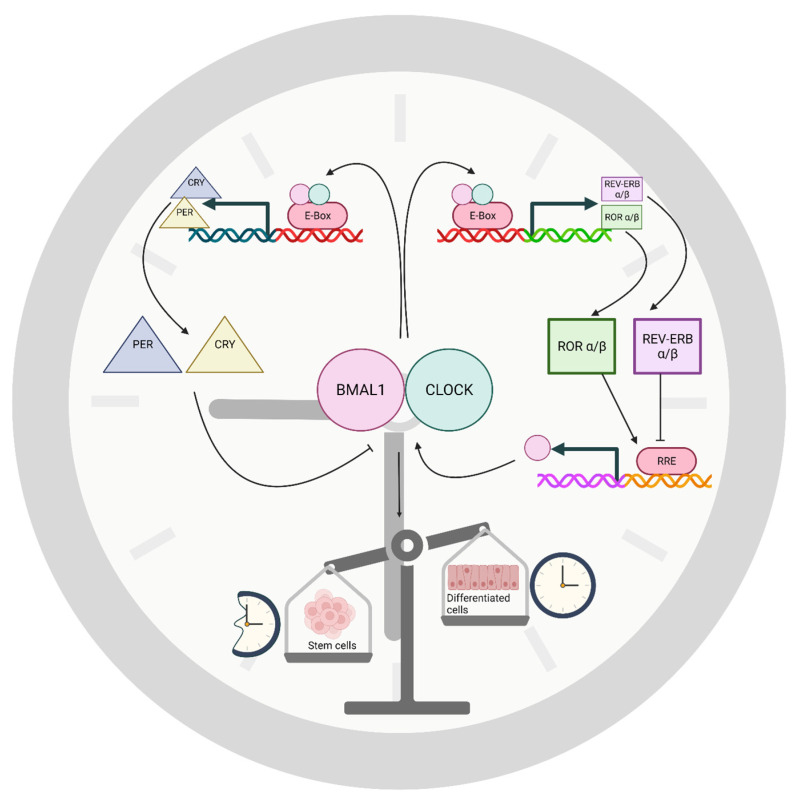
The basic circadian clock timing mechanism of mammals. Two feedback loops work together to provide approximately 24-h rhythms in interacting transcription, translation, and gene regulation. The oscillations simultaneously modulate clock-controlled genes that generate rhythms outside this core timing mechanism. A dimer of transcription factors BMAL1 and CLOCK activates genes through the E-box and related promoter elements. Members of the Per and Cry gene families are, thereby, induced by BMAL1-CLOCK. Resulting PER and CRY proteins enter the nucleus and, through a larger complex, inhibit BMAL1-CLOCK activity, suppressing their own genes but only after a sufficient delay to produce the period of the circadian rhythm. A second loop consists of Rev-erb and ROR genes that are induced by BMAL1-CLOCK, ultimately inducing the Bmal1 gene through a competitive interaction of transcription factors at the RORE of the Bmal1 gene. A generalized view of circadian timing during cell differentiation suggests that circadian timing is suppressed in stem cells, indicated by the broken clock, followed by emergence of a functional clock in progenitor cells and during cell maturation.

The oscillatory regulation of circadian gene expression is carried out by interlocked transcriptional-translational feedback loops (TTFLs) requiring around 24 h for completion. Through these molecular pathways, clock genes activate transcription of their repressors, thereby regulating their own expression (Figure 1). In mammals, the TTFL consists of a core loop and stabilizing loop. In the core loop, the transcription factors CLOCK (Circadian Locomotor Output Cycles Kaput) and BMAL1 (Brain and Muscle Arnt-like protein) heterodimerize and bind to E-box enhancer elements to activate transcription of Cryptochrome (Cry) and Period (Per) genes. Once Per and Cry have been translated, they dimerize and shuttle back into the nucleus. Once in the nucleus, the PER/CRY dimer within a larger protein complex inhibits transcription induced by Clock and BMAL1. Thus, Per and Cry transcription decreases, followed by reduced PER and CRY protein expression. Once PER and CRY levels are low enough, following PER and CRY degradation, the inhibition of Clock and BMAL1 activity is lifted, and a new cycle of transcription begins. This process takes approximately 24 h, and thereby drives the circadian rhythm. In a second, stabilizing loop, ROR and REV-ERB proteins, both regulated by the same E-box as Per and Cry, competitively bind to the RORE promoter site, which rhythmically regulates Bmal1 transcription. Bmal1 is induced by ROR binding and repressed by REV-ERBα [19,20].

The circadian clock regulates cycles of gene expression via transcriptional, post-transcriptional, and post-translational modifications and indirectly generates circadian rhythms in behavior and physiology. BMAL1 circadian rhythms are a major regulator of clock-controlled genes (CCGs), producing rhythms in numerous cellular processes through this clock output [10]. Circadian rhythms are self-sustained; they persist in experimental or natural conditions that do not provide external timing cues for the clock. During entrainment, however, these circadian oscillators repeatedly shift their phase so that their period matches that of the Zeitgeber. Through entrainment, cells produce a rhythmic gene expression pattern suitable for the environment [21]. Circadian oscillations are therefore cell intrinsic but also mutable allowing them to maintain harmony with the local environment and to maintain organization of the individual circadian clocks within the timing system of the organism.

To adapt to cyclical daily changes, organisms have an internal timing system that proactively regulates a huge variety of physiological processes including the sleep/wake cycle and rhythms in metabolism, cell division, and immune activity. Circadian clocks are present in most cells of the body including the various types of stem cells. Although intriguing, their role in the timing of stem cell homeostasis, differentiation, and many other aspects is just beginning to be explored. About 40% of protein-coding genes are under direct or indirect circadian clock control and these include regulators of cell stemness properties [22]. Furthermore, evidence indicates the transitions between stem cell states are rhythmically modulated by the clock, as described below. Herein, we will first provide an overview of stem cell properties and then further discuss the current literature on circadian clock regulation of embryonic and neural stem cells. These properties will also be examined in cancer stem cells (CSCs), which are considered initiators of tumors and tumor recurrence following cancer treatment. Mesenchymal stem cells (MSCs), also known as mesenchymal stromal cells, which differentiate into cells of osteoblast, chondroblast, and adipocyte lineages, will also be examined [23].

### 1.3. Stem Cells

Stem cells are essential for the development of tissues during embryogenesis and they enable homeostasis, growth, and regeneration of tissues during postnatal stages. These undifferentiated cells, also known as the primal cells, give rise to multiple cell lineages. Differentiation after fertilization produces pluripotent stem cells that generate the ectoderm, endoderm, and mesoderm germ layers, which differentiate further into tissues of the developing fetus. Embryonic stem cells (ESC), derived from the inner cell mass of the blastocyst, are unspecialized cells essential for development. These pluripotent stem cells continue to divide and differentiate into multipotent stem cells that can only produce cells of specific lineages, for example, hematopoietic or neural stem cells [8,24].

## 2. Embryonic Stem Cells and Circadian Rhythms

**Figure 2:**
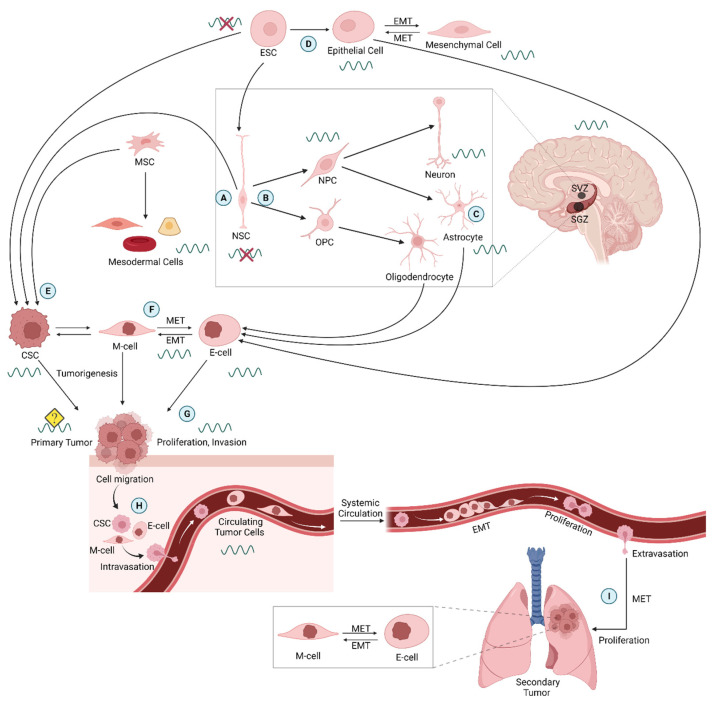
Circadian properties of stem cells and their lineages during development and cancer. Circadian rhythms have been identified at several cell stages (indicated by sine waves); although, evidence during some critical steps remains lacking. See text for additional explanation. (**A**) Circadian rhythms appear to be absent in ESCs and NSCs but emerge as cells differentiate into NPCs and then mature cells. (**B**) Circadian rhythms have been described in differentiating ESCs, adult NSCs, and immature neurons (neuroblasts) in culture [29]. (**C**) Intrinsic circadian rhythms have been identified in a great many mature cell types including neurons and astrocytes [30]. The possibility of circadian rhythms in oligodendrocytes and OPCs has been evaluated; although, evidence is not yet conclusive [31,32]. (**D**) Additional exploration is needed to identify possible circadian rhythms in EMT or MET during development or in non-cancerous adult stem cells such as during growth and tissue repair. Circadian rhythms in melatonin levels may modulate EMT [33,34,35]. (**E**) CSCs may originate directly from adult NSCs, ESCs, or possibly MSCs [36,37]; although, evidence of circadian control of this process is lacking. NSCs of the subventricular zone of the lateral ventricles can generate glioblastomas [38]. (**F**) CSCs also result from M-cells through dedifferentiation of E-cells during EMT, which is reversed by MET. Studies have shown control of EMT by core circadian clock genes [39,40,41]. Circadian clocks in cancer cells control EMT events in glioma and breast cancer cells [42]. Nevertheless, it is not yet known whether M-cells are rhythmic. Any M-cells formed from MSCs, through EMT or possibly mesenchymal cell transformation, are presumed to be rhythmic based on rhythms in the closely related CSCs. Circadian rhythms have been observed in CSC cultures [43,44]. M-cells transition into CSCs, but already have some stem cell characteristics. (**G**) Circadian rhythms have been observed in glioma, lung carcinoma, melanoma, and other cancer cell cultures primarily consisting of E-cells; although, low numbers of M-cells or CSCs can be present [45,46,47]. Circadian rhythms in tumor growth and cell proliferation have been reviewed recently [11,48]. Circadian rhythms described in tumors appear to vary with cancer type. Circadian clocks within tumors generally appear to be dampened, suppressed, or disorganized [11]. The role of circadian rhythms in infiltration of tumor stromal cells and control of the tumor microenvironment and metastasis has been described [49], suggesting that the circadian clock controls migration in the initial stages of metastasis. (**H**) CTCs may have circadian properties because they include CSCs and E-cells of certain cancers that express circadian rhythms. The cell phenotype may change because of their new microenvironment while in circulation. Evidence suggests CTCs can respond to TGF-β that induces EMT, and CTCs may proliferate while in the bloodstream [50]. The number of prostate CTCs shows a daily rhythm that also persists in mice in constant darkness [50]. Note that unlike other tumors considered here gliomas rarely metastasize [51], and the cellular environment of brain capillaries is more complex than indicated here. (**I**) Before formation of secondary tumors, CSCs may migrate, followed by differentiation to the E-cell phenotype through MET at a new location [52]. Circadian rhythms in the surrounding tissue may affect secondary tumors and may differ from the site of origin. M-cell: mesenchymal cancer cell; E-Cell: epithelial cancer stem cell; CSC: cancer stem cell; CTC: circulating tumor cell; MSC: mesenchymal stem cell. NSC: neural stem cell; ESC: embryonic stem cell. NPC: neural progenitor cell; OPC: oligodendrocyte progenitor cell; EMT: epithelial–mesenchymal transition. MET: mesenchymal–epithelial transition.

No coherent information is available on whether a functional circadian clock exists in pluripotent stem cells such as ESCs and, if so, what role the circadian clock might play in these cells during development. Previous studies have demonstrated that clock genes are expressed before activation of the zygotic genome, although they are unlikely to serve in functional circadian feedback loops and a clock in early developmental stages. This idea was conferred when Yagita et al., 2010, showed that undifferentiated mouse ESCs did not show circadian rhythms in bioluminescence when the promoter of the clock gene Bmal1 (Arntl) was used to express firefly luciferase [25]. When these cells were differentiated toward a neural fate, circadian oscillatory bioluminescence was present, indicating NSCs derived from ESCs do have a functioning clock (Figure 2A,B). Furthermore, when the NSCs were induced to dedifferentiate, the cells exhibited loss of circadian oscillations [25]. These findings and others suggest that ESC differentiation is important for establishing circadian clock functioning during brain development, including within the SCN [26,27,28].

Evidence that the circadian clock serves specific functions during early development was found when CRISPR/CAS9-mediated gene editing techniques were used to knockout the gene for CLOCK in mouse ESCs [53]. Although the cells remained pluripotent and able to produce cells of all three embryonic layers, cell proliferation decreased, apoptosis increased, spontaneous differentiation was accelerated, and circadian rhythms were lost in the differentiated tissues.

In vivo mouse studies suggest that there is a cell-intrinsic, independent cellular clock system that regulates embryogenesis, but the more complex, synchronized system is not apparent until after birth [54]. Disruption of the clock gene Arntl (Bmal1) in mice results in overtly normal mice when they are young. Similarly, newborn mice that lack clock genes exhibit normal epidermal wound healing, while adults with the same knockout exhibit severe wound healing defects [55]. Preliminary studies demonstrated that there are circadian rhythms of energy consumption observed as oscillations in glucose utilization and transcription of glucose transporter mRNA [56]. This interesting result suggests that the circadian TTFL may not be necessary for rhythmic activity of some basic processes within the embryonic stem cell.

## 3. Neural Stem Cells and Circadian Rhythms

### 3.1. Characteristics and Functions

During neurogenesis within the embryo and throughout development and adulthood, NSC self-renewal and differentiation maintains the population of proliferating neural progenitor cells (NPCs) that differentiate to form neurons and astrocytes, along with a parallel pathway producing oligodendrocytes (Figure 2C). Two areas of the adult mammalian brain where neurogenesis is perhaps best understood are the subventricular zone (SVZ), located just outside the lateral ventricles, and the subgranular zone (SGZ) of the hippocampal dentate gyrus. Both regions have neurogenic niches but the type of NSCs they contain are different. In the SVZ, the neurogenic cell population consists of type B cells, which express Nestin and glial fibrillary acidic protein (GFAP) and act as quiescent NSCs. When they initiate cell division, they give rise to type C progenitor cells. These cells rapidly divide to differentiate into either neuroblasts, type A cells expressing polysialylated neural cell adhesion molecule (PSA-NCAM), or glia (oligodendrocytes and astrocytes). Type A cells form long chains toward the olfactory bulb, and they become integrated as periglomerular or granule neurons within the existing neuronal environment [57]. In the SGZ, the neurogenic niche is comprised of quiescent type 1 radial glial-like cells, which act as NSCs. These cells generate type 2 cells, which can be classified further into type 2a and 2b cells. Type 2a cells exhibit characteristics of radial glia, such as Nestin and SOX2 expression, but are morphologically distinct. Type 2b cells express Nestin, while also expressing markers of neuronal lineage such as Doublecortin (DCX), PSA-NCAM, or NeuroD. Both subsets of type 2 cells are progenitors that give rise to type 3 cells (neuroblasts). Upon further differentiation of type 3 cells, immature neurons will arise, which will eventually become granule neurons [58].

### 3.2. Circadian Regulation of Adult Neural Stem Cells

The circadian clock appears to play a significant role in adult neurogenesis. Goergen et al., 2002, first discovered diurnal rhythmic neurogenesis in the crustacean brain. Proliferation was observed to peak around dusk, the time at which crustacean brains are most active, suggesting that neurogenesis is primarily regulated by a light-dependent mechanism [59]. A similar mechanism was later discovered in the mammalian hippocampus [60], as was the expression of clock genes in the SVZ and SGZ [61,62]. In vitro studies using neurosphere cultures from mouse SVZ identified a circadian clock 3–4 days after differentiation was induced suggesting that the circadian clock is suppressed when cells are in the neural stem cell state. Furthermore, NPCs from the SVZ of adult mice demonstrated a self-regulatory clock mechanism; the cells were able to produce a circadian rhythm without input from a known circadian pacemaker [29].

Few in vivo experiments have drawn a connection between clock genes and neurogenesis. Bouchard-Cannon et al., 2013, discovered the expression of clock genes in the adult mouse SGZ, and quiescent NPCs expressed clock components and proliferated rhythmically [61]. This study also demonstrated the necessity of PER2 and BMAL1 in neurogenesis. Without PER2, entrance of NPCs into the cell cycle from quiescence was dysregulated. Upon genetic ablation of Arntl1, coding for BMAL1, queiscent NPCs were highly proliferative and exhibited a delayed cell cycle exit. Similarly, Rev-erbα knockout mice had increased proliferation and loss of diurnal rhythmicity in the SGZ demonstrating dependence of neurogenesis on clock genes.

It is known that Per2 plays an important role in the circadian clock, yet its role in neurogenesis is less clearly defined. Borgs et al., 2009, reported a higher density of proliferating NPCs and an increased number of immature newborn neurons in the DG [63]. There are differing reports on whether the role of PER2 in neurogenesis is independent of its role in the circadian clock loop. While Borgs et al. observed constant expression levels of Per2 mRNA and PER2 protein in proliferating NPSCs through differentiation into newborn neurons in the murine adult DG, Bouchard-Cannon et al., 2013, reported rhythmic expression of Per2 in type 1 cells of the DG and weak expression in type 2a cells [61]. The circadian rhythm in type 1 cells (NSCs) does not agree with differentiation of NSCs being required before TTFLs and the clock can function. Some questions raised by these studies are: Is PER2’s role in neurogenesis independent of its role in the circadian clock loop, and is its rhythmically fluctuating expression required for neurogenesis?

### 3.3. Additional Influences on Neural Stem Cell Circadian Clocks

Not surprisingly, the outcome of circadian studies can depend on the age of the subjects. For example, significantly increased proliferation was observed in NPCs from 5-week-old Bmal1 and Per2 knockout mice [61], but not in NPCs from 8-week-old Bmal1 knockout mice [64], when compared with wild type mice. Moreover, BMAL1 knockout mice aged 10–15 weeks exhibited significantly reduced overall proliferation of NPCs in the DG. These findings suggest that the hyperproliferation of NPCs seen in younger Bmal1 KO mice may be short-lived, and it might actually induce depletion of NPCs in adult mice. Accelerated aging is a known phenotype of Bmal1 KO mice [65]. This aging could potentially be explained by the decreased population of proliferative NPCs in the adult DG, as the precursor cell population may age more rapidly.

The zebrafish, with its decentralized circadian clock organization, has not been studied extensively in the realm of clock function. Recent investigations have elucidated the expression pattern of clock genes in zebrafish. In the adult zebrafish brain, clock genes are expressed in the neurogenic regions of the brain, as well as globally in a rhythmic fashion [14]. Weger et al., 2013 demonstrated circadian clock activity in the adult zebrafish telencephalon in vivo by utilizing a transgenic zebrafish line that expresses luciferase as a reporter gene under regulation of the circadian E-box enhancer. When luciferase expression was mapped, it was apparent that NPCs of the telencephalon exhibit a circadian rhythm [14]. This reporter model could be useful in mammalian studies, especially when examining multiple cell types and locales. It may provide insight into the role of clock genes in neurogenesis, particularly if this is independent of their role in the clock mechanism.

As discussed previously, the circadian clock regulates many functions necessary for homeostasis and survival. When studying the relationship between the clock and stem cells, it is important to consider the other systemic processes and cues under circadian clock regulation that could provide for cell plasticity and persistence.

### 3.4. Circadian Clock Plasticity in Neurons and Glial Cells

Circadian clocks are found in a diverse range of organisms from bacteria to mammals living all over the world from the tropics to subarctic regions. This ubiquitous distribution emphasizes the adaptive significance of circadian clocks. It has been shown that the adaptation of the circadian clock to the light–dark cycle and seasonal changes depends on the plasticity of the SCN neural network [66]. Clock neurons of *Drosophila melanogaster* undergo circadian remodeling of neuronal processes that is accompanied by changes in the number of synapses and rhythmic changes in cell membrane excitability that are essential for maintaining synapse functioning [67]. This structural remodeling depends on the oscillation of a neuropeptide, pigment dispersing factor (PDF) [67]. Further, it has been shown that a switch in chloride transport and GABAA receptor signaling is essential for the seasonal synaptic plasticity of SCN neurons [68].

Although circadian control of synaptic plasticity is maintained by individual neurons, it results from a collaboration between neurons and glial cells [69]. Cell-intrinsic circadian clocks in these two descendants of NSCs are critical for rhythms in the SCN and in multiple other brain areas [12] (Figure 2C). Studies are also beginning to reveal the role of circadian rhythms in oligodendrocytes and their generation [31,70]. Glial cells are reported to have a significant impact on circadian adaptation of organisms [32,71]. McCauley et al., 2020, reported that pyramidal neurons in the hippocampal area CA1 change their surface expression of NMDA receptors, and astrocytes alter their proximity to synapses by retracting their processes. This collaborative mechanism contributes to temporal dynamics in cognitive processing [72]. Further, it has been shown that an integrated network of neuronal and glial oscillators is key for circadian adaptation of the *Drosophila* visual system [73]. It has been recently reported that astrocytes contribute to rearrangement of pre- and postsynaptic elements responsible for Hebbian mechanisms of plasticity [74]. Artiushin and Sehgal (2020) described astrocytes as the most strongly implicated glial cell type with many of its functions contributing to sleep. In addition, they report that astrocytes are the only glial cell type known to affect circadian oscillators and clock neuron outputs [71].

Oligodendrocyte progenitor cells (OPCs) of the central nervous system comprise another glial subtype that is mitotically active and multipotent (Figure 2B). They differentiate to replenish myelin within the CNS [75]. Evidence indicates OPCs of developing and adult brain also undergo neuronal differentiation [76]. However, the presence of a circadian clock in oligodendrocytes and their progenitors has yet to be demonstrated. Colwell and Ghiani (2020) recently provided evidence that oligodendrocytes and their progenitors likely contain a cell-autonomous circadian clock functioning in temporal regulation of gene expression important for maturation and myelination; although, definitive proof is still needed [70].

Additionally, a circadian pattern of OPC proliferation in the hippocampus that differs from neurogenesis in the subgranular zone (SGZ) was reported by Matsumoto et al. (2011) [70]. Accordingly, OPC proliferation showed a peak in cells in S-phase during the mouse resting period and M-phase peak in the active period, whereas SGZ neurogenesis only exhibited the M-phase peak in the active period. This variation might be due to different types of cell-cycle regulatory proteins that are present in the two types of cells. For instance, cyclin D1 is expressed in hippocampal OPCs, whereas cyclin D2 is present in SGZ neural stem cells. Moreover, cyclin D1 is under the regulation of the Per2 gene, which indicates that the OPC proliferation might be under clock control. This rhythmic OPC proliferation could alter remodeling of neural circuits contributing to synaptic plasticity that is important for hippocampal function [70].

Furthermore, Huang et al. (2020) have shown that circadian rhythms are disrupted in demyelinating lesions, causing the release of the circadian clock-derived signaling molecules secreted frizzled related protein 1 (SFRP1) and SFRP5. These signals result in reduced BMAL1 levels within the SVZ causing adult SVC NSCs to differentiate into oligodendrocyte lineage cells. This sequence of events then restores myelin in the demyelinating lesion. These results indicate possible circadian clock control of oligodendrogenesis in the SVZ. The authors also described how this disruption of normal circadian timing serves a beneficial effect on healing in the brain rather than being a detriment.

OPCs proliferate, migrate, and differentiate into mature oligodendrocytes, which then extend branching processes, express myelin protein, and produce cell membranes to provide myelination within the CNS [75]. Hence, inducing OPC proliferation, migration, and differentiation are potentially crucial therapeutic approaches for treating demyelinating diseases such as multiple sclerosis [77]. Accordingly, Tang et al. (2021) recently reported that exercise by running can reverse depression-like behavior in mice exposed to unpredictable chronic stress by increasing oligodendrocyte differentiation and myelination in the CA1 subfield of the hippocampus [78]. Evidence indicates OPCs also undergo neuronal differentiation [76]. Adult neurogenesis from OPCs has been reported in vitro and in the cerebral cortex in vivo [79,80].

## 4. Circadian Clocks Acting in Tumorigenesis and Metastasis

### 4.1. Cancer Cells within the Circadian Timing System

Most organs in the mammalian body contain their own circadian clock at the cellular level, which is coupled to the overall circadian timing system through endocrine, metabolic, neuronal, and immunological pathways. Many, but not all, individual cells typically contain circadian clocks that can function independently through robust and self-sufficient pacemakers that, as described earlier, depend on an internal core circadian timing mechanism built from TTFLs [81,82]. The circadian system also includes cells with oscillators that are less stable and remain rhythmic for only one cycle before damping out owing to absence of signals from other pacemakers in the system, contrasting with the high degree of self-sufficiency present in the master circadian pacemaker of the SCN [82]. In cancer research, several studies have shown that increased cancer risk and aggressiveness are linked to chronic disruption or weakening of circadian rhythms in the body or cancer cells [83,84,85]. Disrupted daily rhythms in sleep, melatonin levels, immune activity, and other factors have been associated with initiation and increased proliferation of tumors. However, this generalization may be oversimplified and not apply to all cancer types, some of which may benefit from circadian timing [86]. Furthermore, melatonin production is severely suppressed in several laboratory mouse strains [87,88], but these mice are not reported to have an elevated incidence of spontaneous tumors and appear to be resistant to tumor promotion [89].

Tumors harbor heterogeneous cell populations, and cancer cells vary considerably in their degree of differentiation. Although circadian rhythms measured in tumors have been reported to be weak or absent [90], a subpopulation of cancer cells with persistent rhythms may remain undetected within tumors. In support of this possibility are several gene expression studies, primarily relying on PCR or luciferase-based reporter genes, which revealed distinct circadian rhythms in several cancer cell lines including rat glioma C6 [45], mouse lung carcinoma LLC [46], rat adrenal phaeochromocytoma PC-12 [91], human colon carcinoma HCT-116 [92], mouse melanoma B16 [92], and human osteosarcoma U20S [93], along with cell lines derived from human glioblastomas [43,94]. Furthermore, mouse colon tumor cells utilize circadian clock mechanisms for iron uptake [95]. Colon-26 cells show slower proliferation when the core circadian clock gene CLOCK is mutated indicating that some cancer cells benefit from the core clock proteins, perhaps independent of circadian rhythms. Cell proliferation in leukemia stem cells also depends on a cell intrinsic circadian clock [86].

Preliminary studies have explored the potential role of the circadian clock in regulating the dedifferentiation program utilized by aggressive and invasive tumor cells to metastasize from primary to secondary sites and initiate recurrent tumors. These studies examined whether cell-intrinsic circadian clock machinery controls various cellular events and behaviors during the dynamically reversible epithelial–mesenchymal transition (EMT) in tumor cell cultures, thus rhythmically generating a subpopulation of multi-drug resistant and tumor-initiating CSCs, as recently reviewed [96]. In both rat glioma (C6) and human breast cancer (MCF-7) cell lines, EMT events and generation of CSCs were found to occur rhythmically and more effectively at a particular phase of the circadian cycle [42].

Tumorspheres are aggregates of primarily CSCs that mimic the tumor microenvironment and serve as an appropriate model with potentially greater relevance to patient treatments than cell monolayer cultures. Cells in tumorspheres are in a 3-dimensional culture and they interact within subregions varying in hypoxia like those of tumors. C6 tumorspheres in culture clearly show persistent rhythms in core clock gene expression [44]. MCF-7 cells that have aggregated and formed tumorspheres also display robust circadian rhythms, but they lack easily detectable circadian rhythms in gene expression when grown as monolayers under standard cell culture conditions [42]. Other studies showed that manipulating expression of core circadian clock genes interferes with EMT, supporting a place for these molecular pathways in either clock control of EMT or a non-clock role in the absence of a functional clock [97]. Recently, Per2 overexpression in cervical cancer cells was found to suppress the PI3K/AKT pathway used in EMT [98]. Because of the large variation between cancer cells, it is not possible, at present, to state how widespread clock control is during this important event. Furthermore, very few studies have examined the role of the circadian clock in EMT of normal, non-cancer cells during development or in adults (Figure 2D).

CSCs also appear to be generated directly from other stem cells (Figure 2E). Some evidence indicates glioblastomas originate from adult NSCs [36]. Whether this process is rhythmically modulated by the circadian clock is not known. In addition, very little is known about the possible presence of functional circadian rhythms in mesenchymal cancer cells (M-cells) produced through EMT that further dedifferentiate into more stem-like CSCs (Figure 2E,F). Intermediate E/M cells also occur through what is described as epithelial–mesenchymal plasticity [99]. It is possible that M-cells can also be produced through transformation of mesenchymal cells such as in sarcomas; although; this needs to be tested.

CSCs vary according to their stemness properties but have been categorized into two states—either dividing cells or non-dividing (quiescent) cells that are also macroautophagic [100]. Increased numbers of M-cells and CSCs are present in more aggressive tumors raising the question of whether cell behaviors such as motility, chemotaxis, matrix metalloprotease secretion, or differentiation into epithelial cells (E-cells) are also rhythmic and under circadian clock control [86,91,101]. Interestingly, cellular coupling of intestinal circadian clocks likely depends on the Wnt-signaling pathway that is also used by stem cells to regulate cell differentiation [101].

The shift to marker gene expression indicating EMT is suppressed by melatonin in AML12 hepatocytes from normal mice [33]. Melatonin appeared to suppress EMT induced by TGF-β, and because melatonin is produced under circadian clock control during the night any ongoing EMT in normal tissues or cancer cells may also be rhythmic. Other evidence also indicates melatonin suppresses EMT in non-cancer cells [33,34,35] and cancer cells [102], along with its other reported anti-cancer effects including regulation of the immune system [102]. A model unifying these observations depicts the circadian rhythm in melatonin suppressing the Warburg effect during the night, thereby driving cancer cells away from glycolysis at this phase while enabling more aggressive cancer cell activity during the day [103]. A link between this model and possible circadian rhythms in EMT events in tumors would be testable by determining whether there are proportionately more M-type cells than E-type cells during the day and whether this is driven by melatonin.

### 4.2. Metastasis, EMT, and Circulating Tumor Cells

Metastasis is one of the hallmarks of Stage IV cancer progression with poor prognosis and limited treatment outcomes. It consists of dissemination of cancer from a primary tumor site to nearby and distant tissue and organ sites. During metastasis, mesenchymal, M, cells produced by EMT continue to dedifferentiate into CSCs, followed by cell “shedding” from the tumor edge at the primary site. These cells then invade parenchyma and migrate to distant tissue locations ultimately initiating recurrent secondary tumors through mesenchymal–epithelial transition (MET) of M-cells and differentiation of CSCs generated post-EMT. Endothelial, E-cells undergoing proliferation and increasing the tumor volume are also released from primary tumors during metastasis and produce secondary tumors (Figure 2G). Even though EMT is probably not required for metastasis [104], many studies have established major regulatory functions of EMT and MET during cancer progression [105,106,107], and EMT is important in models of chemoresistance in mammalian pancreatic and lung metastases [108,109].

Several studies have suggested that cells at the invasive front of such tumors exhibit enhanced potential to readily undergo EMT and enter the invasion-metastasis cascade [106,110,111,112]. According to this model, initiation of metastasis involves a subset of cells undergoing genetic and epigenetic alterations along with phenotypic transitions characterized by loss of apical–basal polarity and reduced adherence to adjacent cells and basement membrane when compared with other cells of the primary tumor [113]. Histological sections have revealed morphologically identifiable regions of tumors with greater CSC content. Interaction of the tumor with the stromal compartment also defines the tumor microenvironment that further contributes to intra-tumor heterogeneity and cellular plasticity [114].

CSCs, M-cells, E-cells, and E/M cell hybrids released from metastatic tumors enter blood vessels, and the resulting circulating tumor cells (CTCs) can initiate metastatic lesions at distant sites (Figure 2H,I) [115,116,117]. CTCs, which include circulating CSCs and more differentiated tumor cells, serve as appropriate diagnostic biomarkers for detection of cancer during early stages as they can be distinctly identified and characterized by molecular techniques from serum biopsies [118,119,120]; although, the definitive marker protein patterns are not yet fully established [121]. Although CTCs express stem cell markers, they include a distinct heterogeneous sub-population of neoplastic cells that have dissociated from an aggressive tumor and migrated without apparently undergoing complete EMT and full conversion into CSCs. Most notably, some CTCs express epithelial cell adhesion molecule (EpCAM), but post-EMT cells do not [122]. Cells use a bi-stable switch formed from double negative feedback loops to switch from EpCAM expression in epithelial cells to its repression during EMT [122]. The switch appears to be maintained through epigenetic modifications and appears similar to the sustained “phenotype switching” of malignant melanocytes triggered by Notch signaling in the epidermal microenvironment, resulting in a persistent state during subsequent migration [123,124].

Computer modeling has described how metastatic cancer cells undergoing EMT are released as individual cells or clumps of about 2–8 cells and that these cells can be considered hybrid cells with somewhat stable epithelial and mesenchymal characteristics [124]. CTCs originating from breast cancers can have as many as 100 cells within a cluster, and the ratio of M-to-E cells changes dynamically [125]. CTC clusters may change their M-cell composition while in circulation because they bind platelets that release TGF-β, which induces EMT. Furthermore, CTCs may proliferate, increasing the size of circulating clumps [125]. Whether the circadian timing system regulates CTC EpCAM surface proteins, M-to-E cell ratios, or clump sizes is not known.

If cell intrinsic or external factors drive tumors to “shed” cells rhythmically, it is possible that CTCs might appear in the circulatory system at a predictable phase of the circadian rhythm with the number present undergoing an oscillation under the influence of circadian timing signals, as has been recently discussed [126]. A recent study using in vivo flow cytometry with mice reported daily rhythms in CTC abundance that also persisted in constant darkness, indicating the rhythm was endogenous and not merely driven by responses to the light cycle [50]. These CTCs were liberated by orthotopic tumors formed from prostate cancer cells. The frequency of CTCs present could have been modified by: (1) a circadian clock process acting to facilitate CTCs entering the blood (intravasation); (2) removal from the blood through extravasation of the CTC via the endothelial cell layer; or (3) by other cellular processes that can remove CTCs from circulation. However, a study of multiple myeloma failed to detect circadian rhythmicity in CTC frequency [127], which might have been because the study used immunocompromised mice, which could have altered the intravascular environment of CTCs [128]. However, CTC clusters may be rhythmic because, by analogy, CSCs appear to gain rhythmic functioning through cell–cell interactions while in spheroid cultures [44].

It would also be beneficial to determine whether rhythmic properties of CTCs are driven by a circadian clock within the cells or by external timing signals originating in the surrounding circadian system. CTCs may, for example, benefit from a protective “temporal niche” when daily immune surveillance is minimal [126]. An early study by Hrushesky et al., 1999, indicated that the most effective phase for initiating tumor formation in mice after cancer cell injection is near the transition from sleep to waking, when darkness begins [129]. A comparable phase for increased metastasis may occur in humans. Understanding the origins of these circadian timing effects could greatly facilitate anti-cancer chemotherapeutic strategies by relying on targeting of the circadian clock mechanism or daily timed drug delivery at the most effective circadian phase.

### 4.3. Cancer Stem Cells and Circadian Rhythms

Clinical relevance of CSCs is well established through several studies that have accumulated evidence showing the potential of CSCs to initiate recurrent tumors by evading immune surveillance and standard anti-cancer therapeutic and surgical approaches, increased invasive and migratory properties to initiate secondary metastases through self-renewal and differentiation into progenitor cells, and enhanced resistance to radiation and chemotherapy [130]. CSCs exhibit slower mitosis and often quiescence followed by differentiation into rapidly proliferating cells resembling the progenitor cells serving in development and adult neurogenesis. Thus, targeting CSCs is highly promising for preventing tumor recurrence following treatments and metastases arising from aggressive or invasive primary tumors. Understanding circadian rhythmicity in the expression of core clock genes and CCGs in CSCs is likely to lead to additional strategies to minimize CSCs based on new drug targets and when during the day they are most available for targeting.

Earlier studies indicated an apparent absence of circadian clock functions in embryonic stem cells [25,55]. One possible explanation is that regulation of cell cycle events by the circadian clock may slow down rapid cell proliferation required during early stages of development. Additionally, other studies indicated a possible lack of effective circadian clock machinery in stem cells until they differentiate into progenitor cells [55,131]. Adult neural stem cells gain a functional circadian clock when they differentiate into progenitor cells [29,132]. Does this imply that normal cells lose their circadian clock functions when they undergo trans-differentiation during early development or adulthood? This possibility would also suggest that circadian rhythmicity of cancer cells might be weakened or lost when they dedifferentiate to form M-cells and further progress to generate CSCs following EMT.

However, many assumptions about normal stem cells or CSCs lacking robustly functioning circadian clocks have been based on experimental approaches that were often limited to detecting circadian rhythmicity of clock gene expression among heterogeneous cell populations such as in tumors. Circadian rhythm assays using sequential sampling of animal tumors taken from multiple animals over time or continuous measurements of bioluminescence signals from tumors or cell cultures can be compromised for the same reasons. Arrhythmic cell subpopulations may have been interspersed with rhythmic cell types, thereby masking the rhythmic signal. Furthermore, rhythmic populations that were not well coupled to each other would have produced a distorted rhythm or no discernable rhythm as they reached their peak at different times. Some circadian oscillators damp after a few or several cycles when not exposed to rhythmic humoral or electrical signals from elsewhere in the circadian system. Essentially, identifying circadian rhythms in bulk measurements of signal from heterogeneous and often poorly synchronized cells is prone to false-negative error. Nevertheless, whole-tumorsphere measurements of astrocytoma and breast cancer tumorspheres indicated adequately coupled and rhythmic cells are present that are most likely composed of CSCs or more differentiated cells according to cell phenotypes characterized by expressed stem-cell marker proteins and qRT-PCR [42,44].

Similar to other CSCs, post-EMT M-cells have altered genetic and epigenetic landscapes, phenotypic heterogeneity, and an increased invasive potential and resistance to anti-cancer drugs. However, the microenvironment is important in cancer progression, and in a recent comparative study of C6 glioma and MCF breast cancer cell cultures the M-cells initially emerging through EMT experienced different circadian environments [42]. C6 cells in culture have distinct circadian rhythms before EMT, whereas MCF cell cultures are generally considered arrhythmic, so the nascent M-cells in the first case are provided an opportunity to couple to a rhythmic population, but not in the second. Cancer cell lines such as these, with different circadian properties, could be useful for characterizing how newly liberated metastatic cells interact with the presence or absence of circadian rhythms in their immediate environment. It is the aggregation of some CSCs into tumorspheres that appears to enable their circadian rhythms [44]. Similarly, cell-coupling or density dependent circadian rhythms have been observed in fibroblast cultures [133]. Other rhythmic factors might help CSCs to remain rhythmic and undergo tumorigenesis. For example, VEGF signaling cascades and components that regulate angiogenesis are under clock control [22].

If CTCs, M-cells, and CSCs benefit from having an intrinsic circadian clock, then the circadian properties of the tissues where they are located could influence their survival, particularly if the cancer cell clock functioning is weak. The strength of circadian rhythms within a particular organ or tissue may determine whether it is “fertile soil” for secondary tumor formation, as indicated for other microenvironmental factors. Extracellular vesicles released from CTCs appear to alter surrounding normal cells increasing chances for secondary tumor formation [134]. Extracellular vesicles might also convey circadian timing signals between cells [135]. In summary, multiple properties may determine whether circadian rhythms can persist in metastasized cancer cells and whether their molecular clock components and CCGs will be viable targets for therapeutic treatments.

### 4.4. Evidence of Circadian Clocks Regulating Stem Cell Properties of Cancer Cells

Circadian timing could have an important role during several important differentiation and dedifferentiation events during which circadian timing could have an important role in cancer. As shown in Figure 2, strong evidence of circadian clock regulation is lacking for many of these important events during cancer initiation and progression. Although evidence suggests that a disorganized or suppressed circadian timing system increases cancer risk, tumor growth, and possibly cancer progression, it remains controversial whether it is a sufficient causal factor on its own [136,137]. It may, of course, compound with genetic diversity, mutations, tumor promoters, chronic inflammation, etc.

It is possible that the circadian rhythms in innate and adaptive immune functions determine when cancers are most likely to engage with immune cells [138]. Furthermore, sleep disruption and other disturbances of the circadian timing system might favor cancer cell proliferation or the transitions between stem cell states during EMT or MET at specific times of day; although, strong evidence is lacking. Altered melatonin levels in the body because of excessive nocturnal light exposure to the retina or disrupted circadian timing is a potential contributor to cancer because of melatonin’s anticancer properties [102]. Sensitivity to chemotherapeutic agents has been shown to vary with the phase of circadian oscillations in core clock gene expression of cancer cell cultures [139,140,141].

It is not known whether cells in a pre-cancerous state express circadian rhythms and whether they resemble rhythms in the surrounding tissue. It appears likely that if they lack adequate internal timing capabilities, they will continue to be driven by rhythmic endocrine or neural rhythms in their environment.

## 5. Conclusions

The role of circadian timing in early developing nervous systems, adult neurogenesis, and cancer progression is becoming clearer. Initial views that circadian clocks do not function early in development or in some cancer cells continue to hold, but recent confirmation of circadian rhythms in CSCs tempers that perspective and compels additional consideration of cancer cell and stem cell diversity. A clearer understanding may depend on distinguishing evidence of functioning circadian clocks from results merely showing elevated expression of specific genes used in the circadian timing mechanism that may serve important functions independent of timing.

A great variety of stem cells and stem-like cells are under current consideration by researchers, indicating the complexity of how clock gene expression may integrate with specific cell functions. CSCs, for example, vary across a continuum of phenotypes; although, they distinctly differ transcriptionally and morphologically from the non-CSCs that make up the bulk of tumors. Hybrid EMT cells with epithelial and mesenchymal properties have also been reported [142]. Nevertheless, mesenchymal-type cancer cells are clearly distinguishable from their epithelial-like precursors, yet they share only some of the characteristics of CSCs, and unlike CSCs, their circadian properties are largely untested. Similarly, little is known about the role of circadian rhythms in sarcomas or their stem-like cells. Another fundamental question yet to be adequately addressed is whether specific molecular events of cell differentiation are timed by the clock.

Circadian rhythms and their TTFLs emerge in differentiating embryonic or adult neural stem cells, yet it is difficult to evaluate the earliest, least differentiated cells of these lineages. The challenges arise from the small numbers of neural stem cells and their positions in multiple brain niches while being surrounded by differentiated cells, all of which is not conducive to circadian studies that require monitoring just these cells selectively for days. Embryonic stem cells, although initially abundant, are vigorously active cells that are, again, not favorable subjects for examining stable circadian rhythms. Mathematical approaches more suitable for non-stationary rhythms such as wavelet analysis [143] may prove valuable in exploring circadian properties of these dynamic and transient cell types. Quiescent cancer stem cells and tissue-resident somatic stem cells prior to their activation seem to be better candidates for measuring circadian rhythms.

In vitro studies may provide more satisfying answers, being devoid of confounding rhythms originating elsewhere in the organism and where individual stem cells and their descendants may be reliably tracked. The complexity of interacting risk factors for carcinogenesis and cancer progression, including disturbed circadian timing, can be selectively eliminated and tested in combination though gene editing and pharmacology. Nevertheless, properties of the intact organism are of highest importance for understanding how the circadian system evolved and functions in healthy individuals and during cancer. Organoids are, of course, a promising model for precise circadian studies and have already yielded discoveries with intestinal stem cells undergoing development [144]. Similarly, tumorsphere cultures from at least two cancer types revealed robust circadian rhythms in these densely packed structures [42]. Nevertheless, like the tumors they represent, the specific degree of stemness of the cells generating circadian rhythms in tumorspheres is not yet known.

Advances relevant to these questions will likely depend on studies of the many cells in differentiation and dedifferentiation lineages that have yet to be examined. Included would be cells undergoing EMT in normal tissues or cancers, migrating stem cells, CTCs, and OPCs along with their resulting myelinating cells. Additional explorations initiated with the many stem cells available may reveal the benefits provided by circadian clocks in maintaining temporal order during metabolism, mitosis, dedifferentiation, and other cellular processes operating with a predictable rhythm.

## Abbreviations

SCN	Suprachiasmatic nucleus
TTFL	Transcription translation feedback loop
CLOCK	Circadian locomotor output cycles kaput
BMAL1	Brain and muscle arnt-like protein
PER	Period
CRY	Cryptochrome
CSC	Cancer stem cells
MSC	Mesenchymal stem cells
ESC	Embryonic stem cells
NSC	Neural stem cells
SVZ	Sub ventricular zone
SGZ	Sub granular zone
OPC	Oligodendrocyte progenitor cells
EMT	Epithelial to mesenchymal transition
MET	Mesenchymal to epithelial transition
CTC	Circulating tumor cells
M cells	Mesenchymal cancer cell
E cells	Epithelial cancer stem cell
